# CUSUM: A tool for early feedback about performance?

**DOI:** 10.1186/1471-2288-6-8

**Published:** 2006-03-02

**Authors:** Winston R Chang, Ian P McLean

**Affiliations:** 1 From the Department of Trauma & Orthopaedics, Dumfries and Galloway Royal Infirmary, Bankend Road, Dumfries, DG1 4AP, UK

## Abstract

**Background:**

Modern day clinical practice demands evidence justifying our choice of treatment methods. Cumulative sum techniques (cusum) are amongst the simplest statistical methods known. They provide rapid analysis and identification of trends in a series of data. This study highlights use of these techniques as an early performance indicator of a clinical procedure before its implementation.

**Methods:**

Twenty consecutive patients who underwent total hip or knee arthroplasty received a simple dressing – blue gauze and Tegaderm. Cusum charting was used to assess the dressing with regards to skin blistering. At an acceptable level of performance the curve would oscillate about the horizontal axis and the overall trend therefore said to be flat. If performance is unacceptable, the cusum slopes upward.

**Results:**

The cusum plot for the twenty patients did not cross the specified control limits. This showed that our simple dressing met specified standards with regards to wound blistering postoperatively.

**Conclusion:**

We recommend the use of this simple, yet versatile cusum technique in the early evaluation of a clinical procedure before its implementation.

## Background

The practice of medicine has evolved through time to the current era of evidence based practice. Medical audit is thus vital to any clinical practice. Systematic approaches to peer review of medical care should be encouraged in order to identify opportunities for improvement and provide a mechanism for realising them. Therefore, some form of objective monitoring, or quality control, of practices or procedures is needed so that periods of suboptimal performance in relation to an agreed standard can be recognised and, ideally, remedied.

The use of the cumulative sum (cusum) has been suggested for both surveillance and quality control [1]. First described by Page in 1954 [2], they were applied later to medical problems, replicability of urea estimations and cough remedies by 1965 [3], and were advocated for medical use by Healy in 1968 [4]. Cusum plots may be performed on any data gathered serially. Their main use is in quality control in medical laboratories and industry. Recent experience with this simple yet versatile and powerful statistical technique has amply confirmed its utility, and it is my hope that this study, as an example, will encourage and lead to its wider use in orthopaedics.

We had noted a recent increase in postoperative wound blisters following joint arthroplasty in our District General Hospital. There was a variable practice amongst each orthopaedic unit with regard to postoperative wound dressings. A decision to develop a protocol was then taken. A simple dressing consisting of blue gauze and Tegaderm was used in a preliminary clinical trial as part of a stepwise introduction. Our aim was to ensure that it was at least as effective in early clinical outcome in reducing postoperative wound blisters to acceptable levels, whilst subjecting as few patients as possible should it prove to be unsatisfactory.

## Methods

### Statistical analysis

Minitab 14 (Minitab version 14, Minitab Inc., State College, PA, USA) was used for all statistical and graphical analysis.

### Cusum

A cusum chart is basically a graphical representation of the trend in the outcomes of a series of consecutive procedures performed over time. It is designed to quickly detect change in performance associated with an unacceptable rate of adverse outcome. At an acceptable level of performance, the cusum curve runs randomly at or above a horizontal line (no slope). However, when performance is at an unacceptable level, the cusum slope changes.

For a series of observations X_1_, X_2_, ......... X_n_, the cusum can be defined as

S_n _= Σ(X_0 _- X_i_)

Where X_i _= 1 for a success and X_i _= 0 for a failure. X_0 _is a reference or target value set for the level of performance. A success of nine out of ten would have a target value of 0.9. In practice, this means that for every failed attempt the cusum increases by an increment of 0.9 and each success reduces the cusum by 0.1 [6].

For example, in a series consisting of a success followed by a failure and four successes, the cusum would take the values -0.1, 0.8, 0.7, 0.6, and 0.5. By summing the deviation from the process target in this way, positive and negative deviations will tend to cancel each other out and the cusum plot will run horizontally when the system is stable. If the system average begins to change, the plot will move increasingly upwards or downwards. The deviation will become apparent quickly and this rapid response is a feature of cusum charts and their use.

Although the cusum chart is inspected visually to detect any change in slope, methods to decide when control limits have been exceeded are well described and quite straightforward. One such method involves the superimposition of a (truncated) '*V*-mask' [7].

**Figure 1:**
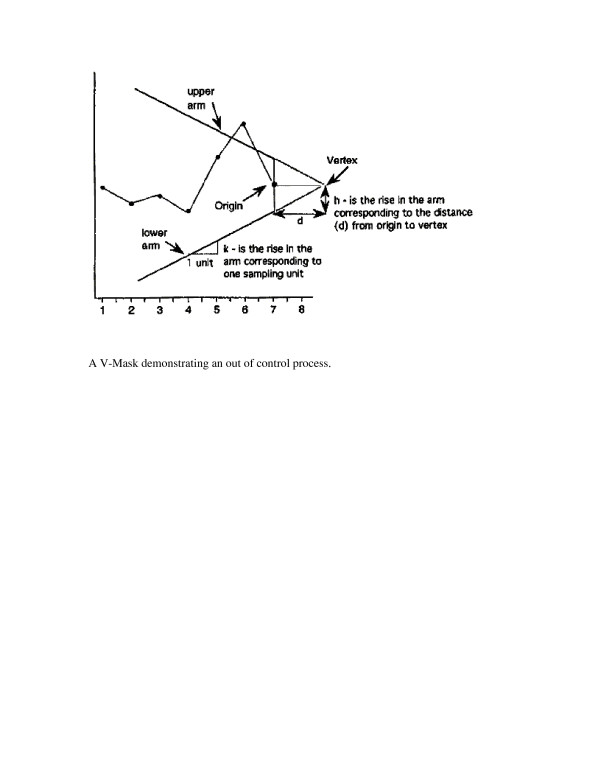
A *V*-Mask demonstrating an out of control process.

A *V*-Mask is an overlay shape in the form of a *V *on its side that is superimposed on the graph of the cumulative sums. The origin point of the *V-*Mask (see Figure 1 below) is placed on top of the latest cumulative sum point and past points are examined to see if any fall above or below the sides of the *V*. As long as all the previous points lie between the sides of the *V*, the process is in control. Otherwise (even if one point lies outside) the process is suspected of being out of control.

From Figure 1, it is clear that the behaviour of the *V*-Mask is determined by *k *(which is the slope of the both arms) and the rise distance *h*. These are the *design parameters *of the *V*-Mask. A detailed description of the construction of the V-mask is beyond the scope of this article, but is mathematically outlined in the statistical literature [7-9].

In general, an appropriate value for the control limit, h, in any specific example is based on the desired average run length (ARL) of the cusum while the failure rate is acceptable. The average run length is equal to the number of patients seen before the cusum first exceeds the control limit. Ideally if the surgical failure rate has not changed (and is acceptable), the run length is long because signals then would represent false alarms. On the other hand, if the failure rate has increased substantially, short run lengths are desirable to ensure remedial action is brought about in a timely fashion. Thus, a narrow V-mask will detect change more quickly but it will give more frequent false alarms. On the other hand, we could reduce the frequency of false alarms by widening the V-mask, but the average run length for real changes would be increased. Hence, h and k must be set so as to detect any real change quickly but in such a way that an interruption is unlikely if no real change has occurred [10] The ARL for differing values of h and k is obtained from the use of a table or nomogram. [11]

A general rule of thumb for the standard cusum is to choose *k *to be half the amount of shift in the process mean that we wish to detect, expressed as a multiple of the standard deviation of the data points and *h *to be around 4 or 5. Commonly recommended alternatives to the standard cusum scheme are (h = 8, k = 0.25) when a higher sensitivity is required for very small shifts or slow trends or if larger shifts or faster trends (h = 2.5, k = 1) is desired. [12]

For the purpose of this study a standard V-mask was created using Minitab software where h is equal to 5 standard deviations and k is equal to 0.5 of a standard deviation of the data points. The average run length for this was around 465 when the dressing performance was acceptable. Given the frequency of hip and knee joint arthroplasty our district hospital, this implies one false positive signal from the monitoring procedure on average every 18 months. Differences greater than 1 standard deviation gave an ARL of 10 which would be detected within a period of one to two weeks. If surgical procedures were more frequent, it might be desirable to select a longer average run length while the surgical mortality rate is acceptable.

### Design of the trial

Twenty consecutive patients who underwent total hip or knee replacement were studied. All operations were performed by the same author. A simple dressing consisting of blue gauze and Tegaderm was used. Each patient's named nurse scored their wound. This was done at the first change of dressing on the third post-operative day followed by daily scoring thereafter by the same nurse on each occasion until discharge.

The incidence of postoperative wound blistering has not been previously reported in the literature [13]. Previous audits on total hip replacement undertaken on our unit in the past showed a 10% incidence of wound blisters. This was therefore adopted as the acceptable rate of wound blisters which gave a target value, X_0 _= 0.9. Hence, a successful outcome i.e. a wound without a blister by the time of discharge would score -0.1. A blistered wound scored +0.9.

## Results

**Figure 2:**
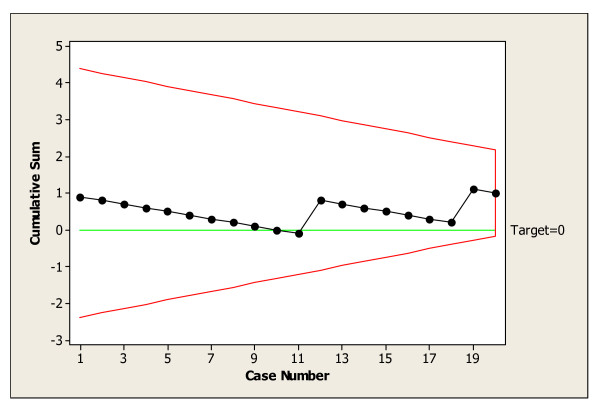
Cusum plot for joint replacement wound blisters using blue gauze and Tegaderm as the wound dressing.

The results for the twenty patients were recorded as shown in Table 1 (see additional file 1). The cusum chart for this is given in Fig. 2.

The first patient in the trial had developed a wound blister. Subsequent wound blistering occurred in the twelfth and nineteenth patient respectively. However, the cusum plot for the twenty consecutive patients was flat. This was confirmed by the use of a standard *V*-mask generated by Minitab software. This indicated that the performance of our simple dressing, blue gauze and Tegaderm, met our specified standards with regards to wound blistering postoperatively.

## Discussion

Clinical medicine still involves much guesswork with consequences that may be fraught with drama and disappointment for patient and practitioner alike. In our efforts to improve this via clinical research too much emphasis has been placed on randomised control trials. Some believe that it is the only valid method for comparing treatments. A closer look however, reveals many drawbacks e.g. ethical considerations, duration of RCT's, substantial resources and funding, and difficulty in blinding, just to highlight a few [14]. Hence, the need for study types other than randomised trials should be recognised.

One of these, the plotting of cumulative sums (cusum) has proved particularly valuable. Its use for examining sequential measures or for detecting changes over time has been described in the past [15]. It has also been used for plotting temperature charts for assessing antimicrobial treatment in neutropaenic patients [16]. More recently, they have been applied as a means of assessing surgical skills of trainees [6].

Our study demonstrates another possible use of the cusum. It allowed us to assess the early performance of a simple dressing in a preliminary trial before developing a departmental protocol on wound dressings. A randomised control trial may take at least four years for a single surgeon to recruit enough patients for a trial of reasonable power to compare two different dressings (estimated 140 patients in each group). In addition RCT's consume substantial resources and are therefore not justified for some questions about small modifications to treatment.

Another, advantage for using a quality control procedure is that after each observation it is possible to make one of two decisions: to accept that the level of performance is satisfactory or to conclude that it is not up to standard; a decision made if the cusum rises above a certain boundary line on the plot.

Choice of control limits needs careful consideration because serious differences in outcomes may go undetected with inappropriate set limits. Similarly, performance could be within acceptable standards yet false alarms are generated purely due to random variation in outcomes. False alarms can be tolerated provided there is a mechanism for doing so and provided that they are not too frequent. If their frequency is a cause for concern, then the control limits can be set higher, thereby increasing the number of patients before a false positive signal. However, caution is needed, as this can mean longer delays before a genuine signal, during which time unnecessary patient injury may occur. Ideally, false alarm rates should be low whilst true alarms are signaled early. In practice this is difficult to achieve most times and a consensus on what is acceptable for true and false alarm rate needs to be agreed upon before setting control limits.

Although not much has been written about postoperative wound blistering in the literature, there is an association noted between the type of dressing used and the incidence of wound blisters [13]. Our preliminary study utilising the cusum allowed us to verify in a relatively short period that the early performance of blue gauze and Tegaderm met our criteria i.e. an acceptable rate of wound blisters of less than 10 percent with regard to wound dressing. With such evidence, we were thus able to incorporate blue gauze and Tegaderm as part of our wound dressing protocol.

Continued surveillance using the cusum is important in ensuring that this standard is maintained since it allows early detection of problems that lead to an increased failure rate. This would lead to a review and possibly remedial measures that could prevent unnecessary future failures. In an era of evidence-based medicine, such quality control and objective and quantified recording of the findings meet the recommended criteria for medical audit [17].

## Conclusion

We recommend that this simple cusum technique be considered as a means of evaluating, introducing or testing any new procedure or practice. Early identification of unacceptable standards would therefore be picked up thereby exposing as few patients as possible to any unsatisfactory outcome.

## Competing interests

The author(s) declare that they have no competing interests. No benefits in any form have been received or will be received from a commercial party related directly or indirectly to the subject of this article.

## Authors' contributions

Both authors contributed to the planning, execution and completion of the project. The article was written up by the first author with advice and guidance from the second author.

## Pre-publication history

The pre-publication history for this paper can be accessed here:



## Supplementary Material

Additional File 1 **Table 1**: Record chart for the results of the 20 patients studied. Click here for file

## References

[B1] Williams SM, Parry BR, Schlup MT (1992). Quality control: an application of the cusum. BMJ.

[B2] Hurst HE (1950). Proceedings of American Society Civil Engneers.

[B3] Armitage P (1960). Sequential Medical Trials.

[B4] Healy MJR (1968). Br med Bull.

[B5] Lim TO, Soraya LM, Ding LM, Morad Z (2002). Assessing doctors' competence: application of cusum technique in monitoring doctors' performance. International Journal for Quality in Health Care.

[B6] VanRij AM, McDonald JR, Pettigrew RA, Putterill MJ, Reddy CK, Wright JJ (1995). Cusum as an aid to early assessment of the surgical trainee. Br J Surgery.

[B7] Barnard GA (1959). Control charts and stochastic processes. J Roy Stat Soc Ser B.

[B8] Page ES (1996). Cumulative sum control charts. Technometrics.

[B9] Page ES (1963). Controlling the standard deviation by CUSUMS and warning lines. Technometrics.

[B10] Chatfield C Statistics for Technology.

[B11] Woodall WH, Adams BM (1993). The Statistical Design of CUSUM Charts. Quality Engineering.

[B12] Montgomery DC (2000). Introduction to Statistical Quality Control.

[B13] Gupta SK, Lee S, Moseley LG (2002). Postoperative wound blistering: is there a link with dressing usage?. J Wound Care.

[B14] McCulloch P, Taylor I, Sasako M, Lovett B, Griffin D (2002). Randomised trials in surgery: problems and possible solutions. BMJ.

[B15] Altman DG, Royston P (1988). The hidden effect of time. Statistics in Medicine.

[B16] Kinsey SE, Giles FJ, Holton J (1989). Cusum plotting of temperature charts for assessing antimicrobial treatment in neutropaenic patients. BMJ.

[B17] Shaw CD, Costain DW (1989). Guidelines for medical audit: seven principles. BMJ.

