# Supplementary material for: CUSUM: A tool for early feedback about performance?
Source: BMC Med Res Methodol. 2006 Mar 2;6:8. doi: 10.1186/1471-2288-6-8 (PMC1420317; doi:10.1186/1471-2288-6-8)
Supplement: Additional File 1 — Table 1: Record chart for the results of the 20 patients studied. [file 1471-2288-6-8-S1.doc]

| **Number** | **Blisters** | **Score** | **CUSUM** |
| --- | --- | --- | --- |
| 0  1  2  3  4  5  6  7  8  9  10  11  12  13  14  15  16  17  18  19  20 | Y  Y  Y | 0  0.9  -0.1  -0.1  -0.1  -0.1  -0.1  -0.1  -0.1  -0.1  -0.1  -0.1  0.9  -0.1  -0.1  -0.1  -0.1  -0.1  -0.1  0.9  -0.1 | 0.0  0.9  0.8  0.7  0.6  0.5  0.4  0.3  0.2  0.1  0.0  -0.1  0.8  0.7  0.6  0.5  0.4  0.3  0.2  1.1  1.0 |

**Table 1** Record chart for the results of the 20 patients studied.
